# Predicting severe COVID-19 disease in adults: A single-centre cohort study during the first three pandemic waves in 2020–2021 in Vilnius, Lithuania

**DOI:** 10.1371/journal.pone.0350112

**Published:** 2026-05-29

**Authors:** Ieva Kubiliute, Edgaras Zaboras, Fausta Majauskaite, Jurgita Urboniene, Birute Zablockiene, Giedre Gefenaite, Aukse Mickiene, Ligita Jancoriene

**Affiliations:** 1 Clinic of Infectious Diseases and Dermatovenerology, Institute of Clinical Medicine, Faculty of Medicine, Vilnius University, Vilnius, Lithuania; 2 Faculty of Medicine, Vilnius University, Vilnius, Lithuania; 3 Centre of Infectious Diseases, Vilnius University Hospital Santaros Klinikos, Vilnius, Lithuania; 4 Department of Health Sciences, Faculty of Medicine, Lund University, Lund, Sweden; 5 Department of Infectious Diseases, Lithuanian University of Health Sciences, Kaunas, Lithuania; King Fahd Military Medical Complex, SAUDI ARABIA

## Abstract

**Background:**

Since its emergence, the COVID-19 infection has led to significant morbidity and mortality worldwide. Early identification of patients at risk for severe disease is essential for more effective triage, timely therapeutic intervention, and optimal resource allocation. Differences in population characteristics may contribute to variability in disease outcomes, which emphasizes the need for regional-level data, especially from underrepresented regions. The main aim of this study was to identify the demographic, clinical, and laboratory predictors of severe COVID-19, defined as the need for oxygen therapy, in Lithuania.

**Materials and methods:**

We conducted an ambispective observational cohort study at Vilnius University Hospital Santaros Klinikos in Vilnius, Lithuania, from March 2020 to December 2021. Adult patients with a confirmed diagnosis of COVID-19 and hospitalized longer than 24 hours were included in this study. Data were collected from the electronic medical records and patient interviews. To identify predictors of severe COVID-19 course, a multivariable binary logistic regression model was performed.

**Results:**

Among 495 patients, 52.9% were male, the median age was 55 years, and 61.2% had at least one underlying condition. The most common symptoms on admission were malaise (77.1%), subfebrile fever (65.9%), and cough (69.7%). CRP demonstrated the highest predictive value for severe COVID-19 (AUC = 0.84), followed by LDH (AUC = 0.80). Older age (OR 1.04 per year, 95% CI 1.00–1.08), obesity (OR 3.55, 95% CI 1.35–9.30), lymphopenia (OR 3.70, 95% CI 1.37–9.99), higher LDH (OR 1.008, 95% CI 1.00–1.01) and CRP (OR 1.021, 95% CI 1.01–1.04) levels were identified as the strongest predictors for severe COVID-19 disease course.

**Conclusion:**

Older age, obesity, lymphopenia, and higher CRP and LDH were associated with developing severe COVID-19 disease, indicating that combining patient history and laboratory parameters can provide a practical risk stratification approach to help clinicians identify high-risk patients early upon hospitalisation.

## Introduction

Since its emergence in late 2019, the coronavirus disease 2019 (COVID-19), caused by the severe acute respiratory syndrome coronavirus 2 (SARS-CoV-2), has led to significant morbidity and mortality worldwide. COVID-19 has a wide range of clinical manifestations. While most infected individuals are asymptomatic or experience mild to moderate symptoms, a substantial proportion develop severe or critical illness that is characterized by respiratory failure, multisystem involvement, and deadly complications [1,2]. Early identification of patients at risk for severe disease is essential for appropriate triage, timely initiation of therapy, and optimal resource allocation.

Numerous predictive models have been developed to identify patients at risk of severe disease since the onset of the COVID-19 pandemic. These efforts highlight various demographic, clinical, and laboratory factors like older age, male sex, comorbidities (e.g., cardiovascular disease, diabetes), and specific biomarkers as being associated with worse outcomes [3–6]. However, despite the widespread use of both traditional statistical approaches and advanced machine learning techniques, there is still no clear consensus on a unified set of predictors. Many existing models are limited by limited external validation, heterogeneity in study populations, and methodological constraints, which raises concerns about their generalizability and real-world clinical utility [7–9].

Although the global incidence of severe COVID-19 cases has declined over recent years, especially with the emergence of less virulent variants and increasing immunity through vaccination or prior infection, it remains crucial to understand which clinical factors contributed to the broad spectrum of disease severity. Identifying the predictors of severe COVID-19 can not only inform the management of future SARS-CoV-2 outbreaks but also serve as a reference point for future pandemics caused by novel respiratory pathogens. Early recognition of patients at risk for deterioration enables more effective triage, timely therapeutic intervention, and potentially improved clinical outcomes. For instance, some studies have demonstrated that early warning scores, such as the National Early Warning Score (NEWS), can predict adverse outcomes in COVID-19 patients, facilitating prompt clinical decisions [10,11]. Additionally, research indicates that early identification of high-risk patients allows targeted therapies to be implemented, which may reduce mortality and the need for intensive care [12]. Moreover, this approach could help reduce the overall burden on healthcare systems by enabling resource prioritization, minimizing preventable complications, and supporting tailored treatment strategies.

Despite the proliferation of risk models for predicting COVID-19 severity, many lack external validation and are heavily influenced by local population characteristics, including genetic background and ethnicity, healthcare infrastructure, diagnostic criteria, and other often unassessed variables [7]. The potential variability of these factors highlights the importance of generating country-specific or at least region-specific data. Differences in population characteristics may contribute to variability in disease outcomes across settings, so this underscores the need for regional-level evidence across different countries [13,14]. Regional data from Eastern and Central Europe remain underrepresented in the global literature, even though healthcare systems in these areas faced unique challenges during the pandemic [15,16]. Thus, region or country-specific studies are crucial in identifying the specific predictive variables in this often-understudied European region. Additionally, the clinical utility of many proposed markers varies depending on the timing of presentation, restricted availability of advanced laboratory marker tests outside major tertiary care centres, and thresholds used for clinical decisions [17,18]. In Lithuania, like in many other countries, the early pandemic period was marked by diagnostic uncertainty and evolving treatment protocols [19]. We therefore designed a study to:

1. Describe the baseline demographic, clinical, laboratory, and radiological characteristics, complications, and hospitalization outcomes of adults hospitalized with COVID-19 in Vilnius, Lithuania, during 2020–2021.
2. Evaluate the predictive accuracy of laboratory parameters for a severe COVID-19 disease course.
3. Identify the strongest demographic, clinical, and laboratory risk factors of severe COVID-19, defined as the need for oxygen therapy.
4. Compare clinical, laboratory, and radiological characteristics, complications, and hospitalization outcomes across different COVID-19 waves.

## Methods

### Study design and setting

We conducted an ambispective observational cohort study at Vilnius University Hospital Santaros Klinikos, a tertiary care university hospital in Vilnius, Lithuania, combining retrospectively (13 March – 30 June 2020) and prospectively collected data (1 July 2020–31 December 2021).

### Participants and recruitment

Individuals aged 18 years and older with a confirmed diagnosis of COVID-19 infection who were hospitalized for more than 24 hours in the COVID-19 department of Vilnius University Hospital Santaros Klinikos were included in this study. COVID-19 diagnosis was confirmed by a positive SARS-CoV-2 reverse transcriptase polymerase chain reaction test or a rapid antigen test on a nasopharyngeal swab sample. The antigen test was administered to symptomatic patients within 5 days of the onset of COVID-19 symptoms. Exclusion criteria were hospitalization for ≤ 24 hours, as well as refusal to participate, or inability to communicate and provide informed consent for participants enrolled prospectively. In total, 495 eligible patients were included in the study, of which 258 (52.1%) were included retrospectively and 237 (47.9%) prospectively.

### Data collection and variables

Data were collected from the electronic medical records of Vilnius University Hospital Santaros Klinikos and through direct patient interviews. Both sources provided information about demographic factors, underlying medical conditions, and other clinical data. Information on objective findings, laboratory results on admission, radiological findings, details of in-hospital complications, intensive care unit (ICU) or high dependency unit (HDU) admissions, length of hospitalization, and hospital discharge status (discharged, transferred, or died) were extracted from the medical records.

Demographic variables were age (in years) and sex (male/female). Comorbidities, recorded as binary variables (yes/no), comprised anaemia or other haematological disease, asthma, chronic obstructive pulmonary disease (COPD), other pulmonary disease (excluding asthma and COPD), non-haematological malignancy, arterial hypertension, other cardiovascular disease (excluding arterial hypertension), dementia, diabetes mellitus, immunodeficiency, chronic liver disease (including cirrhosis), neuromuscular disease, chronic kidney disease, rheumatological disease, prior stroke, tuberculosis, obesity, and malnutrition. Additional medical information consisted of smoking status (never smoked/ former smoker/ current smoker), body mass index (BMI; numeric, kg/m²), pregnancy status (yes/no), and history of COVID-19 vaccination (vaccinated/unvaccinated).

The presence of COVID-19 symptoms at admission was recorded as present or absent for each of the following: sudden onset of symptoms, subfebrile fever, febrile fever, chills, tachypnoea, malaise, headache, dizziness, confusion, myalgia, sore throat, coryza, cough, shortness of breath, chest pain, palpitations, general deterioration, nausea, vomiting, diarrhoea, abdominal pain, ageusia, anosmia, conjunctivitis, rash, or other dermatological manifestation.

Collected objective findings on admission were body temperature (°C), respiratory rate (breaths per minute), and oxygen saturation (SpO₂; %). The risk of patients’ clinical deterioration was classified using the National Early Warning Score (NEWS) as low risk (0–4), medium risk (5–6), or high risk (≥ 7).

Initial laboratory test results performed at the time of hospitalization, all treated as numeric variables, and included complete blood count, C-reactive protein (CRP), ferritin, interleukin-6 (IL-6), lactate dehydrogenase (LDH), D-dimer, fibrinogen, alanine aminotransferase (ALT), aspartate aminotransferase (AST), gamma-glutamyl transferase (GGT), alkaline phosphatase (ALP), lactate, troponin I, N-terminal pro-B-type natriuretic peptide (NT-proBNP), creatinine, urea, prothrombin time (PT), and international normalized ratio (INR).

From the medical records, we also obtained information on chest X-ray and computed tomography (CT) findings, development of pneumonia, the required oxygen therapy (through nasal cannula and face mask), high-flow therapy, non-invasive ventilation, invasive mechanical ventilation, and extracorporeal membrane oxygenation (ECMO), all recorded as binary variables.

Furthermore, we collected the data on complications developed during the hospitalization, that included acute respiratory distress syndrome (ARDS), bronchiolitis, secondary bacterial pneumonia (confirmed by positive bronchoalveolar lavage culture), other secondary bacterial infection (confirmed by positive culture), sepsis, acute renal failure, heart failure, multiple organ dysfunction, critical illness myopathy, pulmonary embolism, dermatological complications and any other complication not mentioned above. All complications were recorded as binary variables.

Information about the transmission to the ICU or HDU (yes/no), length of stay in these units, overall length of hospitalization (in days), and hospital discharge status (discharged/transferred, died) was also obtained.

The COVID-19 pandemic in Lithuania in 2020–2021 was marked by distinct periods of SARS-CoV-2 variant dominance: the Alpha variant until September 30, 2020, the Beta variant from October 1, 2020, to July 1, 2021; the Delta variant until December 31, 2021.

The outcome of this study was severe COVID-19 disease, defined as a clinical course requiring any form of oxygen therapy during hospitalization, while non-severe disease was defined as a clinical course in which oxygen therapy was not needed.

### Statistical analysis

Descriptive statistics were used to summarize baseline demographic, clinical, laboratory, and radiological characteristics, complications, and hospitalization outcomes. Numeric variables were presented as medians with interquartile ranges (IQR), while categorical variables were summarized using absolute and relative frequencies.

Group comparisons were conducted to analyse differences between severe and non-severe COVID-19 cases and across COVID-19 waves. The Shapiro–Wilk test was used to assess the normality of continuous variables, and none met the assumption of normal distribution. Therefore, group comparisons for numeric variables were performed using the Mann–Whitney U test or the Kruskal–Wallis test with Bonferroni correction for multiple comparisons. Comparisons for categorical and binary variables were made using the χ² test or Fisher’s exact test, as appropriate. A cumulative distribution curve was used to illustrate the overall distribution of oxygen saturation values.

To address the second study objective, the predictive performance of laboratory parameters was assessed by calculating the area under the receiver operating characteristic (ROC) curve.

To address the third objective – identifying risk factors associated with the development of severe COVID-19 – univariable and multivariable binary logistic regression models were constructed. The univariable models included demographic, clinical characteristics, and laboratory values measured at admission. Variables that were statistically significant in the univariable analysis were reviewed by clinical experts for inclusion in the multivariable regression model. Highly subjective clinical symptoms (e.g., malaise, dizziness, general deterioration, confusion), highly correlated variables (ALT, which was strongly correlated with AST; r = 0.803) were excluded from the multivariable model. Tachypnoea, SpO_2,_ and NEWS were also not included in the model as they directly reflect the need for oxygen therapy. The final multivariable logistic regression model included age, male sex, subfebrile fever, febrile fever, chills, cough, shortness of breath, chest pain, diarrhoea, obesity, COPD, arterial hypertension, other cardiovascular disease (excluding arterial hypertension), lymphocyte count (dichotomized at 1.0 × 10⁹/L), and the following laboratory analytes: neutrophil count, AST, ferritin, IL-6, LDH, D-dimer, and CRP. Odds ratios (OR) with 95% confidence intervals (CI) were reported. Regression model performance was evaluated by assessing explanatory power using Cox & Snell and Nagelkerke R² statistics. Model calibration was examined using the Hosmer-Lemeshow goodness-of-fit test. The classification performance of the model was evaluated using a probability cut-off value of 0.50. Internal validation of the regression model was performed using bootstrap resampling (1000 samples) to assess the stability of the identified predictors.

Missing data were evaluated by examining the proportion of missing values for each variable. Cases with missing data were excluded from group comparisons. Laboratory variables with a high proportion of missing values (> 20%) were excluded from ROC and multivariable regression analyses. For the remaining variables, analyses were performed using available cases.

A p-value < 0.05 was considered statistically significant. All analyses were performed using IBM SPSS Statistics, version 30.0.

### Ethical aspects

This study was approved on July 3, 2020, by the Lithuanian Bioethics Committee (approval number L-20–3/1), with amendments approved on July 25, 2022, January 25, 2023, December 13, 2023, and December 20, 2024. Signing informed consent was waived for retrospectively enrolled participants (from March 13, 2020, to June 30, 2020), whereas from July 1, 2020, to December 31, 2021, only participants who signed the written informed consent were admitted to the current study. During this study period (from July 3, 2020, to December 31, 2021), after obtaining approval from the Lithuanian Bioethics Committee, only the researchers had access to information that could identify individual participants included in the study (retrospectively and prospectively), allowing them to collect the required data in accordance with the study protocol. After this period, once the necessary data had been collected, only depersonalized data was used for further analysis.

## Results

### Baseline characteristics

A total of 495 adult patients hospitalized with laboratory-confirmed COVID-19 were included in the study. Of these, 52.9% were male. The median age was 55 years, and 37.4% of patients were aged 60 years or older. Almost two-thirds of the patients (61.2%) had at least one underlying medical condition. The most frequent comorbidities were arterial hypertension (51.5%), other cardiovascular diseases (20.8%), and diabetes mellitus (11.1%). The median BMI was 29.7 kg/m^2^. Half of the participants were obese (n = 137/271, 50.6%). While information on smoking was missing for 257 participants, among those assessed, 29.4% were identified as former smokers, and 7.1% as current smokers. Almost 6% of patients were vaccinated against COVID-19 (Table 1).

**Table 1:** Baseline characteristics of hospitalized COVID-19 patients for the total sample and by disease severity.

Characteristic	N	Total (N = 495), n (%)	Non-severe COVID-19 (N = 204), n (%)	Severe COVID-19 (N = 291), n (%)	p-value
Age in years, median (IQR)	495	55 (43–65)	48 (35–61.75)	58 (48–68)	<0.001
Age groups, years					
18–29	39	39 (7.9)	33 (16.2)	6 (2.1)	<0.001
30–39	56	56 (11.3)	34 (16.7)	22 (7.6)	
40–49	99	99 (20.0)	47 (23.0)	52 (17.9)	
50–59	116	116 (23.4)	34 (16.7)	82 (28.2)	
60–69	105	105 (21.2)	32 (15.7)	73 (25.1)	
70–79	45	45 (9.1)	13 (6.4)	32 (11.0)	
≥ 80	35	35 (7.1)	11 (5.4)	24 (8.3)	
Male sex	495	262 (52.9)	95 (46.6)	167 (57.4)	0.018
Smoking					
Never smoked	238	151 (63.5)	39 (66.1)	112 (62.6)	0.006
Former smoker		70 (29.4)	11 (18.6)	59 (33.0)	
Current smoker		17 (7.1)	9 (15.3)	8 (4.5)	
Obesity	271	137 (50.5)	24 (29.6)	113 (59.5)	<0.001
Any comorbidities	495	303 (61.2)	104 (51.0)	199 (68.4)	<0.001
Comorbidities					
None	495	192 (38.8)	100 (49.0)	92 (31.6)	<0.001
One	495	137 (27.7)	51 (25.0)	86 (29.6)	
Two or more	495	166 (33.5)	53 (26.0)	113 (38.8)	
Pregnancy (only female)	233	5 (0.02)	0 (0.0)	5 (0.02)	–
Anaemia or any other haematological disease	495	40 (8.1)	13 (6.4)	27 (9.3)	0.243
Asthma	495	17 (3.4)	6 (2.9)	11 (3.8)	0.614
COPD	495	14 (2.8)	1 (0.5)	13 (4.5)	0.009
Other pulmonary diseases, not asthma or COPD	495	11 (2.2)	3 (1.5)	8 (2.8)	0.266
Non-haematological oncological disease	495	28 (5.7)	8 (3.9)	20 (6.9)	0.159
Arterial hypertension	495	255 (51.5)	83 (40.7)	172 (59.1)	<0.001
Other cardiovascular disease (not arterial hypertension)	495	103 (20.8)	31 (15.2)	72 (24.7)	0.010
Dementia	495	13 (2.6)	5 (2.5)	8 (2.8)	0.838
Diabetes mellitus	495	55 (11.1)	16 (7.8)	39 (13.4)	0.053
Immunodeficiency	495	15 (3.0)	3 (1.5)	12 (4.1)	0.090
Chronic liver disease, including cirrhosis	495	18 (3.6)	9 (4.4)	9 (3.1)	0.440
Neuromuscular disease	495	12 (2.4)	4 (2.0)	8 (2.8)	0.403
Chronic kidney disease	495	32 (6.5)	10 (4.9)	22 (7.6)	0.236
Rheumatological disease	495	17 (3.4)	5 (2.5)	12 (4.1)	0.314
Prior stroke	495	18 (3.6)	9 (4.4)	9 (3.1)	0.440
Tuberculosis	495	3 (0.6)	3 (1.5)	0 (0.0)	0.069
Vaccinated against COVID-19	495	29 (5.9)	5 (2.5)	24 (8.3)	0.007

Mann-Whitney U test; χ² test or Fisher’s exact test, as appropriate, were used for calculations.

IQR – interquartile range; COPD – chronic obstructive pulmonary disease.

Two hundred ninety-one patients (58.8%) developed severe COVID-19 disease. Severe cases were more often male, about 10 years older, and had statistically significantly higher BMI compared with non-severe cases (Table 1).

Comorbidities were significantly more common among those with severe disease (68.4% vs. 51.0%). COPD (4.5% vs. 0.5%), arterial hypertension (59.1% vs. 40.7%), and other cardiovascular diseases (24.7% vs. 15.2%) were more prevalent among severe cases compared to those with non-severe COVID-19. Diabetes mellitus was also more frequent in the severe COVID-19 disease course group (13.4% vs. 7.8%), though this did not reach statistical significance (Table 1).

### Clinical symptoms

The clinical symptoms reported by patients at the time of hospital admission are compiled in Table 2. Throughout the entire sample, the most frequently reported symptoms were malaise (77.0%), subfebrile fever (65.9%), cough (69.7%), and shortness of breath (40.5%). Most patients (81.5%) experienced the sudden onset of the disease.

**Table 2:** Clinical symptoms of COVID-19 patients at the time of hospitalization by disease severity (symptoms were reported by patients).

Symptoms	N	Total (N = 495), n (%)	Non-severe COVID-19 (N = 204), n (%)	Severe COVID-19 (N = 291), n (%)	p-value
Sudden onset of symptoms	482	393 (81.5)	168 (84.4)	225 (79.5)	0.171
Subfebrile fever	495	326 (65.9)	122 (59.8)	204 (70.1)	0.017
Febrile fever	495	179 (36.2)	46 (22.6)	133 (45.7)	<0.001
Chills	490	143 (29.2)	41 (20.4)	102 (35.3)	<0.001
Tachypnoea	495	91 (18.4)	7 (3.4)	84 (28.9)	<0.001
Malaise	488	376 (77.1)	124 (62.0)	252 (87.5)	<0.001
Headache	488	152 (31.2)	66 (33.0)	86 (29.9)	0.461
Dizziness	488	85 (17.4)	15 (7.5)	70 (24.3)	<0.001
Confusion	491	24 (4.9)	5 (2.5)	19 (6.6)	0.036
Myalgia	488	142 (29.1)	61 (30.5)	81 (28.1)	0.570
Sore throat	488	101 (20.7)	50 (25.0)	51 (17.7)	0.051
Coryza	488	73 (15.0)	30 (15.0)	43 (14.9)	0.983
Cough	489	341 (69.7)	126 (63.0)	215 (74.4)	0.007
Shortness of breath	489	198 (40.5)	36 (18.0)	162 (56.1)	<0.001
Chest pain	488	114 (23.4)	32 (16.0)	82 (28.5)	0.001
Palpitations	488	41 (8.4)	13 (6.5)	28 (9.7)	0.207
General deterioration	492	151 (30.7)	37 (18.2)	114 (39.5)	<0.001
Nausea	488	54 (11.1)	18 (9.0)	36 (12.5)	0.225
Vomiting	495	12 (2.4)	4 (2.0)	8 (2.8)	0.769
Diarrhoea	491	62 (12.6)	15 (7.5)	47 (16.2)	0.004
Abdominal pain	488	28 (5.7)	11 (5.5)	17 (5.9)	0.851
Ageusia	487	75 (15.4)	25 (12.5)	50 (17.4)	0.139
Anosmia	487	109 (22.4)	44 (22.0)	65 (22.7)	0.866
Conjunctivitis	495	11 (2.2)	4 (2.0)	7 (2.4)	1.000
Rash/other dermatological manifestation	495	4 (0.8)	1 (0.5)	3 (1.0)	0.646

Mann-Whitney U test, χ² test, or Fisher’s exact test, as appropriate, were used for calculations.

Patients who developed severe COVID-19 disease were significantly more likely to present with general malaise, cough, fever, shortness of breath, and tachypnoea. Other symptoms that were also more common in severe cases included chills, chest pain, dizziness, confusion, general deterioration, and diarrhoea. Symptoms such as headache, myalgia, sore throat, coryza, palpitations, nausea, vomiting, ageusia, and anosmia did not differ significantly between these two groups (Table 2).

Regarding timing, patients with severe COVID-19 were admitted to the hospital later after the onset of symptoms compared to the non-severe patient group. The median time from symptom onset to hospitalization was 7 days (IQR: 4–9) in the severe group and 4 days (IQR: 2–8) in the non-severe group (p < 0.001).

### Objective clinical findings and laboratory parameters on admission

Patients with severe disease had slightly higher body temperature (36.9°C vs. 36.8°C, p = 0.025) compared with patients in the non-severe group. The median respiratory rate was similar between groups (both 16 breaths/min), but the distribution was significantly broader in the severe group (p < 0.001). Additionally, the NEWS, a composite indicator of clinical deterioration, was notably higher in the severe COVID-19 group (median 3 vs. 1, p < 0.001).

**Fig 1:**
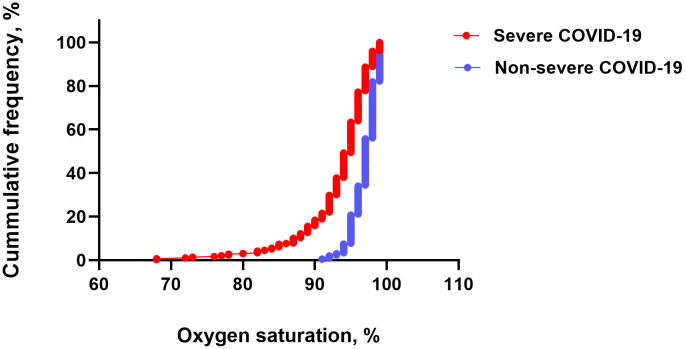
Cumulative distribution of oxygen saturation on admission by COVID-19 severity.

The median oxygen saturation on admission was significantly lower in the severe COVID-19 group (95%, IQR: 92–96) compared to the non-severe group (97%, IQR: 96–98; p < 0.001), and the leftward shift of the cumulative distribution curve in the severe COVID-19 group demonstrated consistently lower oxygen saturation values across the entire distribution (Fig 1).

At the time of hospitalization, among patients who later developed severe COVID-19, 84.2% were classified as low risk based on a NEWS score, 11.7% as medium risk, and 4.1% as high risk. All patients with non-severe COVID-19 on admission were classified as low risk.

On admission, inflammatory markers were substantially higher in patients who developed a severe COVID-19 disease course. Median CRP levels were over nine times higher in the severe group (56.0 vs. 6.2 mg/L), and IL-6 levels were more than three times higher (33.4 vs. 9.9 ng/L). The concentrations of other inflammatory markers, such as ferritin (480.0 vs. 215.0 µg/L), fibrinogen (5.4 vs. 4.4 g/L), were also significantly higher in the severe COVID-19 course group.

Differences in haematological parameters at the time of hospitalization included a lower lymphocyte count (1.05 vs. 1.40 × 10⁹/L) and higher neutrophil count (4.14 vs. 3.30 × 10⁹/L) in the severe group. Total leukocyte and platelet count did not differ significantly between the groups.

There were also other laboratory analytes that concentrations were statistically significantly higher among severe cases: LDH (303.0 vs. 206.0 U/L), AST (38.0 vs. 24.0 U/L), and ALT (34.0 vs. 24.0 U/L), lactate (1.4 vs. 1.1 mmol/L), D-dimer (425.0 vs. 265.0 µg/L), fibrinogen (5.4 vs. 4.4 g/L,), troponin I (8.0 vs. 3.8 ng/L), NT-proBNP (170.3 vs. 98.0 ng/L), urea (5.0 vs. 4.3 mmol/L) and creatinine levels (80.0 vs. 74.4 µmol/L). All analysed laboratory findings are detailed and presented in Table 3.

**Table 3:** Laboratory characteristics of hospitalized COVID-19 patients on admission by disease course.

Variable	Total (N = 495)		Non-severe COVID-19 (N = 204)		Severe COVID-19 (N = 291)		p-value
	N	Median (IQR)	N	Median (IQR)	N	Median (IQR)	
WBC, x10^9^/L	481	5.6 (4.3–7.1)	193	5.6 (4.4–6.9)	288	5.6 (4.2–7.4)	0.601
Neutrophils, x10^9^/L	481	3.8 (2.7–5.2)	193	3.3 (2.4–4.6)	288	4.1 (2.8–5.7)	<0.001
Lymphocytes, x10^9^/L	481	1.2 (0.8–1.6)	193	1.4 (1.1–1.8)	288	1.1 (0.8–1.4)	<0.001
Platelets, x10^9^/L	481	187 (149–236)	193	190 (160–243)	288	182 (144–232)	0.097
ALP, U/L	392	64.0 (52.0–80.3)	139	65.0 (53.8–81.0)	253	63.0 (51.0–84.5)	0.288
ALT, U/L	443	30.0 (18.0–48.0)	169	24.0 (15.0–37.8)	274	34.0 (21.5–56.0)	<0.001
AST, U/L	430	33.0 (24.0–50.0)	156	24.0 (19.2–33.8)	274	38.0 (29.0–64.3)	<0.001
Ferritin, µg/L	398	358.7 (168.6–775.5)	131	215.0 (97.4–409.2)	267	480.0 (227.3–1,113.7)	<0.001
IL-6, ng/L	401	25.0 (11.0–50.6)	128	9.88 (4.9–22.4)	273	33.40 (16.9–63.0)	<0.001
LDH, U/L	407	269.0 (208.0–348.6)	140	206.4 (179.9–252.7)	267	303.0 (249.5–393.0)	<0.001
D-dimer, µg/L	395	375.0 (215.0–640.0)	126	265.0 (145.0–521.3)	269	425.0 (267.5–695.0)	<0.001
Fibrinogen, g/L	360	5.1 (4.2–6.1)	117	4.4 (3.6–5.5)	243	5.4 (4.6–6.3)	<0.001
CRP, mg/L	479	29.9 (6.8–84.1)	193	6.2 (1.9–21.3)	286	56.0 (25.6–112.0)	<0.001
Lactate, mmol/L	367	1.3 (1.0–1.8)	110	1.1 (0.8–1.5)	257	1.4 (1.0–1.9)	<0.001
Troponin I, ng/L	387	6.6 (3.0–13.0)	124	3.8 (2.0–8.8)	263	8.0 (4.0–16.0)	<0.001
NT-proBNP, ng/L	339	147.2 (71.6–386.0)	109	98.0 (54.6–249.0)	230	170.3 (89.0–429.4)	<0.001
GGT, U/L	391	34.0 (18.0–76.0)	135	21.0 (14.0–58.9)	256	40.5 (22.0–85.1)	<0.001
PT, %	387	90.0 (78.0–104.0)	127	90.0 (77.0–103.0)	260	91.0 (78.0–104.0)	0.630
INR	393	1.04 (0.98–1.11)	128	1.04 (0.99–1.12)	265	1.04 (0.98–1.11)	0.428
Creatinine, μmol/L	467	77.6 (64.0–95.0)	180	74.4 (62.0–87.7)	287	80.0 (65.8–97.4)	0.007
Urea, mmol/L	376	4.7 (3.6–6.4)	125	4.3 (3.3–5.3)	251	5.0 (3.9–6.8)	<0.001

Mann-Whitney U test was used for calculations.

ALP – alkaline phosphatase; ALT – alanine aminotransferase; AST – aspartate aminotransferase; CRP – C-reactive protein; GGT – gamma-glutamyl transferase; INR – international normalized ratio; IL-6 – interleukin 6; IQR – interquartile range; LDH – lactate dehydrogenase; NT-proBNP – N-terminal pro-B-type natriuretic peptide; PT – prothrombin time; WBC – white blood cell.

Reference values: WBC: 4.0–9.8 x10^9^/L; neutrophil count: 1.5–6.0 x109/L; lymphocyte count: 1.0–4.0 x10^9^/L; platelets: 140–450 x10^9^/L; ALP: 40–150 U/L; ALT: ≤40 U/l; AST: ≤40 U/L; ferritin: 25–350 µg/L (for men), 13–232 µg/L (for women); IL-6: 0–7 ng/L; LDH: 125–243 U/L; D-dimer: < 250 µg/L; fibrinogen: 2–4 g/L; CRP: < 5 mg/L; lactate: 0.50–2.20 mmol/L; troponin I: < 19 ng/L; NT-proBNP: for acute heart failure suspicion – < 450 ng/L (for <50 years old); < 900 ng/L (for 50–75 years old); < 1800 ng/L (for >75 years old), for chronic heart failure suspicion – < 125 ng/L; GGT: ≤36 U/L; PT: 70–130%; INR: 0.90–1.19; creatinine: 64–104 µmol/L (for males), 49–90 µmol/L (for females); urea: 2.5–7.5 mmol/L.

### Radiological findings and oxygen therapy

Radiological imaging was performed for the majority of patients during hospitalization (Table 4). Among those who underwent chest x-ray, pulmonary infiltrates were significantly more common in patients with severe disease (90.9% vs. 50.5%). Pleural effusion (9.8% vs. 1.6%) and pulmonary stasis (17.5% vs. 4.3%) were also more frequently detected in the severe COVID-19 group.

**Table 4:** Instrumental investigation findings, oxygen therapy, complications, and hospital discharge status of COVID-19 patients by disease course.

Characteristic	Total (N = 495), n (%)	Non-severe COVID-19 (N = 204), n (%)	Severe COVID-19 (N = 291), n (%)	p-value
Chest X-ray	473 (95.6)	187 (91.7)	286 (98.3)	<0.001
Infiltration	354 (75.0)	94 (50.5)	260 (90.9)	<0.001
Pleural effusion	31 (6.6)	3 (1.6)	28 (9.8)	<0.001
Infiltration opacity > 50%	45 (18.4)	0 (0.0)	45 (39.1)	<0.001
Stasis	58 (12.3)	8 (4.3)	50 (17.5)	<0.001
Chest computed tomography	126 (25.5)	49 (24.0)	77 (26.5)	0.539
Peripheral opacity	65 (51.6)	19 (38.8)	46 (59.7)	0.022
Ground-glass opacity	82 (65.1)	27 (55.1)	55 (71.4)	0.061
Consolidation	31 (24.6)	8 (16.3)	23 (29.9)	0.085
Infiltration	62 (49.2)	18 (36.7)	44 (57.1)	0.025
Pleural effusion	15 (11.9)	2 (4.1)	13 (16.9)	0.031
Pneumonia	395 (82.1)	111 (58.4)	284 (97.6)	<0.001
Unilateral	60 (15.2)	31(27.9)	29 (10.2)	<0.0010
Bilateral	335 (84.8)	80 (72.1)	255 (89.8)	
**Oxygen therapy**	291 (58.8)	0 (0.0)	291 (100.0)	<0.001
Nasal cannula	87 (17.6)	0 (0.0)	87 (29.9)	<0.001
Face mask	218 (44.0)	0 (0.0)	218 (74.9)	<0.001
High-flow therapy	29 (5.9)	0 (0.0)	29 (10.0)	<0.001
Non-invasive ventilation	5 (1.0)	0 (0.0)	5 (1.7)	0.081
Mechanical ventilation	14 (2.8)	0 (0.0)	14 (4.8)	0.001
ECMO	3 (0.6)	0 (0.0)	3 (1.0)	0.272
**Complications**				
Any complication	114 (25.1)	10 (4.9)	104 (35.7)	<0.001
Acute respiratory distress syndrome	46 (9.3)	0 (0.0)	46 (15.8)	<0.001
Bronchiolitis	1 (0.2)	1 (0.5)	0 (0.0)	0.412
Secondary bacterial pneumonia (confirmed by positive bronchoalveolar lavage culture)	10 (2.0)	0 (0.0)	10 (3.4)	0.007
Other secondary bacterial infection (confirmed by positive culture)	60 (12.1)	5 (2.5)	55 (18.9)	<0.001
Sepsis	18 (3.6)	0 (0.0)	18 (6.2)	<0.001
Acute renal failure	18 (3.6)	1 (0.5)	17 (5.8)	0.002
Heart failure	5 (1.0)	0 (0.0)	5 (1.7)	0.081
Multiple organ dysfunction	14 (2.8)	0 (0.0)	14 (4.8)	0.001
Dermatological complications	3 (0.6)	1 (0.5)	2 (0.7)	1.000
Critical illness myopathy	1 (0.2)	0 (0.0)	1 (0.3)	1.000
Pulmonary embolism	8 (1.6)	0 (0.0)	8 (2.8)	0.024
Other complication	27 (5.5)	2 (1.0)	25 (8.6)	<0.001
**Transferred to ICU or HDU**	54 (10.9)	0 (0.0)	54 (18.6)	<0.001
Transferred to ICU	49 (9.9)	0 (0.0)	49 (16.8)	<0.001
Transferred to HDU	11 (2.2)	0 (0.0)	11 (3.8)	0.004
**Length of stay**				
Length of stay in hospital in days, median (IQR)	10.0 (7.0–14.0)	8.0 (4.0–11.0)	12.0 (9.0–16.0)	<0.001
Length of stay in ICU in days, median (IQR)	7.0 (5.0–13.0)	0.0 (0.0–0.0)	7.0 (5.0–13.0)	–
Length of stay in HDU in days, median (IQR)	8.0 (4.0–13.0)	0.0 (0.0–0.0)	8.0 (4.0–13.0)	–
Length of stay in ICU and HDU in days, median (IQR)	8.0 (5.0–14.0)	0.0 (0.0–0.0)	8.0 (5.0–14.0)	–
**Hospital discharge status**				
Discharged/Transferred	474 (95.8)	204 (100.0)	270 (92.8)	<0.001
Died during the hospitalization	21 (4.2)	0 (0.0)	21 (7.2)	

Mann-Whitney U test, χ² test, or Fisher’s exact test, as appropriate, were used for calculations.

ECMO – extracorporeal membrane oxygenation; HDU – high dependency unit; ICU – intensive care unit; IQR – interquartile range.

Chest CT was performed in a subset of 126 (25.5%) patients. In the severe group, CT findings more frequently revealed peripheral opacities (59.7% vs. 38.8%), parenchymal infiltration (57.1% vs. 36.7%), and pleural effusion (16.9% vs. 4.1%). Although ground-glass opacities and consolidation were more common in severe cases, these differences were not statistically significant (71.4% vs. 55.1% and 29.9% vs.16.3%, respectively).

A clinical diagnosis of pneumonia was established in nearly all patients with severe COVID-19 (97.6%), compared with 58.4% in non-severe cases, with bilateral pneumonia also significantly more frequent in severe cases (89.8% vs. 72.1%).

The most common oxygen therapy modalities for severe COVID-19 patients included a face mask (74.9%) and a nasal cannula (29.9%). High-flow oxygen therapy was administered to 10.0% of severe patients, and smaller proportions required non-invasive ventilation (1.7%) or mechanical ventilation (4.8%). ECMO was used in 1.0% of severe cases (Table 4).

### Complications and hospital discharge status

Severe COVID-19 cases were associated with a significantly higher complication rate. Over one-third (35.7%) of patients in the severe group experienced at least one complication, compared with 4.9% of those in the non-severe group. The most common complication was other (than secondary bacterial pneumonia) secondary bacterial infection confirmed by positive culture. Overall, it developed in 60 (12.1%) patients and was statistically more common in the severe COVID-19 group (18.9% vs. 2.5%). The second most common complication was ARDS, which developed only in 15.8% of severe cases. Other complications more commonly developed in severe COVID-19 patients compared with non-severe patients were sepsis (6.2% vs. 0.0%), acute kidney injury (5.8% vs. 0.5%), secondary bacterial pneumonia (3.4% vs. 0.0%), and pulmonary embolism (2.8% vs. 0.0%). Multiple organ dysfunction syndrome occurred in 4.8% of severe cases. These and additional complications are detailed in Table 4.

Transfer to ICU/HDU was required for 18.6% of patients in the severe group, while none of the non-severe patients needed such care. The median hospital stay was longer in the severe COVID-19 group (12 days) than in non-severe cases (8 days). Among patients transferred to ICU/HDU, the median length of stay in critical care was 8 days (Table 4).

In-hospital mortality occurred exclusively in the severe group, affecting 7.2% of patients. The discharge/transfer rate was correspondingly lower in the severe group (92.8% vs. 100.0%) (Table 4). Overall, 22 (4.4%) patients were transferred to other facilities for continued care.

### Predictive accuracy of laboratory parameters for severe COVID-19 disease

The predictive performance of initial laboratory tests for identifying patients at risk of developing severe COVID-19 was assessed using ROC curve analysis. The area under the curve (AUC) values for each parameter are presented in Table 5.

**Table 5:** Predictive accuracy for severe COVID-19 of initial laboratory tests performed on admission.

Variable	AUC (95% CI)	p-value
WBC count, x10^9^/l	0.51 (0.46–0.57)	0.601
Neutrophil count, x10^9^/l	0.61 (0.56–0.66)	<0.001
Lymphocyte count, x10^9^/l	0.31 (0.26–0.36)	<0.001
ALT, U/l	0.65 (0.60–0.70)	<0.001
AST, U/l	0.77 (0.72–0.82)	<0.001
Ferritin, µg/l	0.72 (0.67–0.78)	<0.001
IL-6, ng/l	0.77 (0.72–0.82)	<0.001
LDH, U/l	0.80 (0.76–0.85)	<0.001
D-dimer, µg/l	0.65 (0.59–0.71)	<0.001
CRP, mg/l	0.84 (0.80–0.87)	<0.001
GGT, IU/l	0.64 (0.58–0.70)	<0.001
Creatinine, μmol/l	0.57 (0.52–0.63)	0.007

ROC analysis was used for calculations. Sample sizes for every variable are listed in Table 3.

ALT – alanine aminotransferase; AST – aspartate aminotransferase; AUC – area under the ROC curve; CI – confidence interval; CRP – C-reactive protein; GGT – gamma-glutamyl transferase; IL-6 – interleukin 6; LDH – lactate dehydrogenase; WBC – white blood cell.

Reference values: WBC: 4.0–9.8 x10^9^/L; neutrophil count: 1.5–6.0 x10^9^/L; lymphocyte count: 1.0–4.0 x10^9^/L; ALT: ≤40 U/l; AST: ≤40 U/L; ferritin: 25–350 µg/L (for men), 13–232 µg/L (for women); IL-6: 0–7 ng/L; LDH: 125–243 U/L; D-dimer: < 250 µg/L; CRP: < 5 mg/L; GGT: ≤36 U/L; creatinine: 64–104 µmol/L (for males), 49–90 µmol/L (for females).

**Fig 2:**
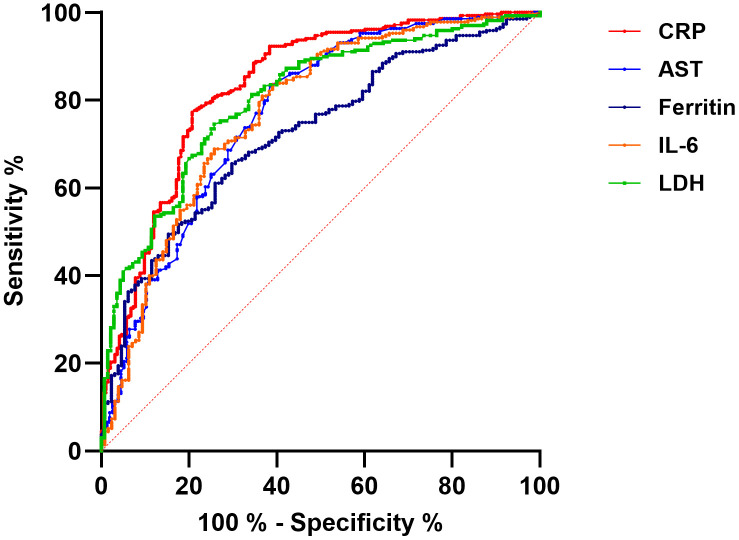
Predictive accuracy of initial laboratory tests on admission for COVID-19 disease severity: ROC curve analysis. AST – aspartate aminotransferase, CRP – C-reactive protein, IL-6 – interleukin 6, LDH – lactate dehydrogenase.

Among the biomarkers analysed, CRP demonstrated the highest predictive value, with an AUC of 0.84, followed by LDH (AUC = 0.80), IL-6 (AUC = 0.77), AST (AUC = 0.77), and ferritin (AUC = 0.72) (Table 5, Fig 2).

### Predictors associated with severe COVID-19 disease course

To identify the key factors associated with severe COVID-19, we performed logistic regression analysis. In univariable analysis, a number of variables showed statistically significant associations with severe disease, including older age, male sex, obesity, arterial hypertension, other cardiovascular disease (not arterial hypertension), COPD, subfebrile fever, febrile fever, chills, tachypnoea, malaise, dizziness, confusion, cough, shortness of breath, chest pain, general deterioration, diarrhoea, lymphocyte count, neutrophil count, ALT, AST, ferritin, IL–6, LDH, D-dimer, fibrinogen, CRP, lactate, creatinine, and urea (S1 Table).

**Fig 3:**
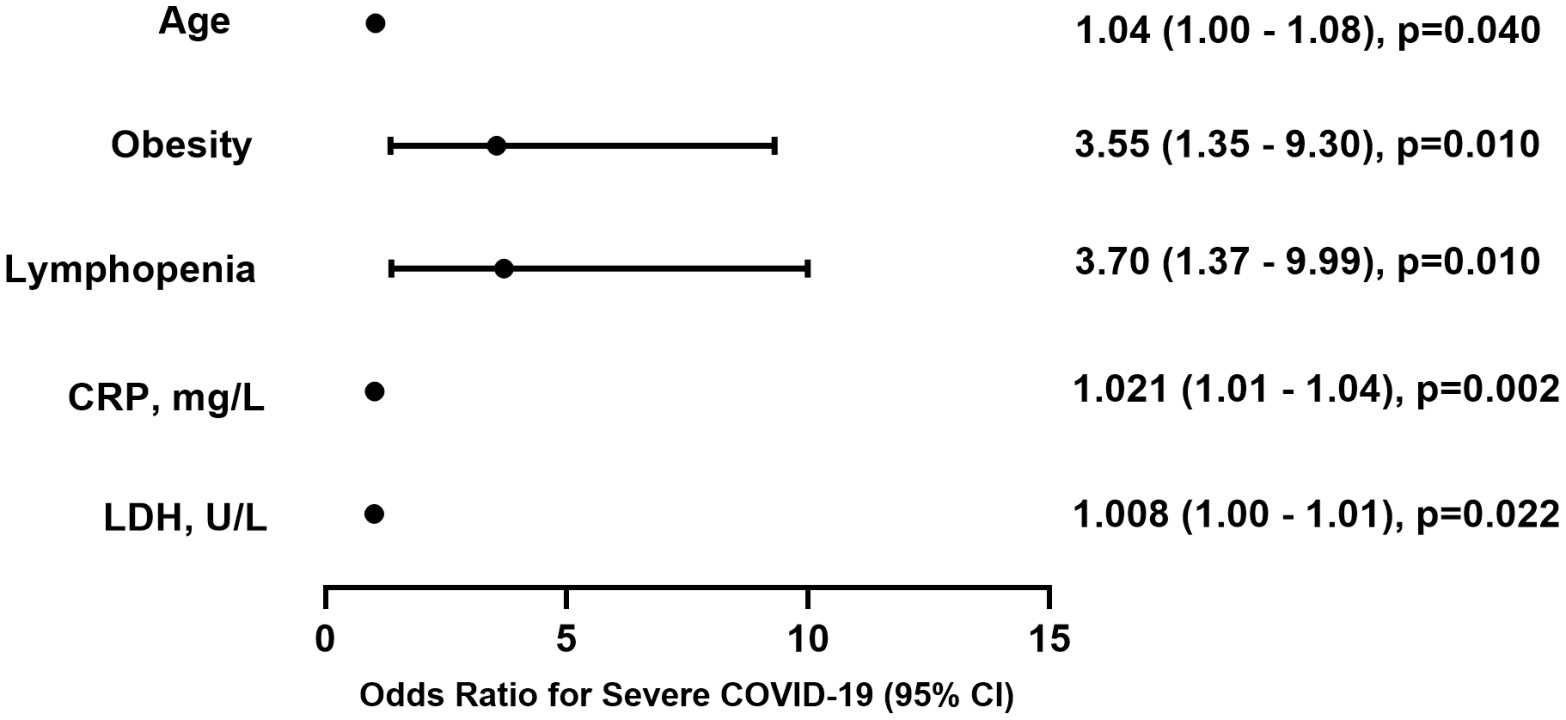
Significant predictors associated with severe COVID-19 disease course, multivariable logistic regression model after internal validation. CI – confidence interval; CRP – C-reactive protein; LDH – lactate dehydrogenase.

In the multivariable model, six predictors were independently associated with the severe COVID-19 disease course, and five remained significant after internal validation (Fig 3). Older age was associated with an increased odds of severe COVID-19 (OR 1.04 per year, 95% CI 1.00–1.08, p = 0.040), while obesity conferred a 3.55-fold higher odds compared with non-obese patients (OR 3.55, 95% CI 1.35–9.30, p = 0.010). Among symptoms at admission, febrile fever was associated with an increased odds of severe COVID-19 (OR 3.41, 95% CI 1.09–10.73, p = 0.036). Lymphopenia on admission was likewise identified as a significant predictor (OR 3.70, 95% CI 1.37–9.99, p = 0.010). In addition, higher levels of LDH and CRP were independently associated with severe COVID-19 (LDH: OR 1.008, 95% CI 1.00–1.01, p = 0.022; CRP: OR 1.021, 95% CI 1.01–1.04, p = 0.002), corresponding to an 8% increase in odds of severe disease per 10 U/L increase in LDH and a 21% increase per 10 mg/L increase in CRP. The model demonstrated acceptable explanatory power (Cox & Snell R² = 0.362; Nagelkerke R² = 0.535). The Hosmer–Lemeshow goodness-of-fit test indicated good calibration (χ² = 3.43, df = 8, p = 0.905). Using a probability cut-off of 0.50, the model correctly classified 83.9% of cases. Internal validation using bootstrap resampling confirmed that age, obesity, lymphopenia, LDH, and CRP remained significant predictors of severe COVID-19 (Fig 3).

### Variation in COVID-19 manifestation across pandemic waves

A total of 337 patients were hospitalized during the Alpha variant wave, 96 during the Beta variant wave, and 62 during the Delta variant wave. The median age of patients did not differ significantly between waves: Alpha 56 years (IQR 43–68), Beta 56.5 years (IQR 47–61), and Delta 50.5 years (IQR 41–60.25) (p = 0.142). The proportion of male patients was 49.3% in the Alpha wave, 62.5% in the Beta wave, and 58.1% in the Delta wave.

Severe COVID-19 disease was diagnosed in 43.6% of patients in wave 1, 93.8% in wave 2, and 87.1% in wave 3. Severe disease was statistically significantly (p-values <0.001) more common in wave 2 and wave 3 as compared with wave 1.

Compared with wave 1, patients in waves 2 and 3 more frequently experienced chills, malaise, dizziness, coryza, cough, shortness of breath, palpitations, general deterioration, and nausea. Additionally, wave 2 patients more often presented with febrile fever and tachypnoea, while wave 3 patients more commonly reported headache, chest pain, vomiting, diarrhoea, ageusia, anosmia, and conjunctivitis. Compared with wave 2, patients in wave 3 more frequently had chills, headache, myalgia, cough, palpitations, anosmia, and conjunctivitis, and less frequently – febrile fever (S2 Table).

Compared with wave 1 patients, initial laboratory test results of patients in waves 2 and 3 showed significantly higher neutrophil counts and lower lymphocyte counts, more pronounced elevations in inflammatory markers such as CRP, ferritin, IL-6, and fibrinogen, as well as higher levels of biomarkers indicating tissue injury or organ dysfunction, including LDH, D-dimer, ALT, AST, GGT, NT-proBNP, and lactate (S3 Table).

Chest imaging findings revealed that patients in waves 2 and 3 had significantly higher rates of radiologically confirmed pneumonia, especially bilateral, compared to wave 1 (p < 0.001). Infiltrates involving more than 50% of the lung fields on chest X-ray were more frequently observed in waves 2 and 3, while ground-glass opacities on chest CT were markedly more common in wave 3 compared to wave 1 (S4 Table).

Patients in waves 2 and 3 more often required oxygen therapy, particularly via face masks, compared with those in wave 1 (p < 0.001). The need for high-flow oxygen therapy was significantly higher in wave 3 than in wave 1 (0.6% vs 14.5%, p < 0.001), while there were no differences between waves 2 and 3 on these parameters (S4 Table).

The incidence of complications – including ARDS, secondary bacterial infections, and PATE – was more frequent in the later waves. ARDS occurred significantly more often in wave 3 than in wave 1 (17.7% vs. 8.9%) and wave 2 (17.7% vs. 5.2%). Non-pulmonary secondary bacterial infections were also more frequent in wave 2 compared with wave 1 (20.8% vs. 9.5%) (S4 Table). Patients were less frequently transferred to ICU/HDU in wave 1 compared with wave 2 (7.7% vs. 15.6%) and wave 3 (7.7% vs. 21.0%), and there were no differences between wave 2 and 3. There were no significant differences in length of stay in the hospital and the result of hospitalization between waves (S4 Table).

## Discussion

In this observational study, we evaluated the demographic, clinical, laboratory, radiological characteristics, and risk factors associated with a severe COVID-19 disease course among hospitalized adult patients during the first three pandemic waves in a tertiary care centre in Vilnius, Lithuania. As one of the few studies from the Baltic region, our findings contribute to a more geographically diverse understanding of COVID-19 heterogeneous pathogenesis and help address the global imbalance in COVID-19 data representation. We identified older age, obesity, lymphopenia, and higher concentrations of CRP and LDH to be independent risk factors for developing a severe COVID-19 disease course described as the need for oxygen therapy. These findings align with a large body of global research demonstrating that age-related and adiposity-driven immune dysregulation, inflammation, the presence of multiple comorbid conditions, and end-organ injury are central to the pathophysiology, progression, and poor outcomes of COVID-19 [3,5,6,20].

The predictive model construction, which includes many patient-specific disease characteristics, laboratory test results, and imaging modality specifics, has not been a new endeavour since the start of the pandemic. Several other clinical studies, even using machine learning techniques, have also aimed to identify key factors associated with COVID-19 severity and outcomes. However, their accuracy has been repeatedly questioned due to many limitations [21–23]. Numerous studies’ predictive models have identified demographic and clinical factors associated with worse outcomes, including older age [24,25], male sex [26,27], obesity [28–34], and the presence of underlying comorbidities such as cardiovascular disease, diabetes mellitus, chronic lung disease (especially COPD) [35–41], immunosuppression [3–6,42], and neuropsychiatric comorbidities [37]. In our study, older age and obesity were the only demographic or comorbidity factors that independently increased the odds of developing a severe COVID-19 disease course. The observed association between older age and severe COVID-19 is consistent with previous evidence showing that advanced age is a major risk factor for severe disease and mortality, likely due to age-related immune dysregulation, including immunosenescence and chronic low-grade inflammation [43–46]. In addition, in a meta-analysis performed by Foldi et al. in 2020, obesity was identified as a significant risk factor for ICU admission (OR=1.21, CI: 1.002–1.46) and for the need of invasive mechanical ventilation (OR=2.05, CI: 1.16–3.64) [30]. Even though in our study other demographic factors and comorbidities, such as male sex, COPD, arterial hypertension, and other cardiovascular diseases, were more prevalent among the severe COVID-19 cases as compared to non-severe cases, they were not statistically significant predictors of severe COVID-19 disease course. Smoking is another factor frequently identified as a risk factor for severe COVID-19 disease [34,37]. In our study, the number of current smokers within the COVID-19 severity subgroups was too small to allow for meaningful statistical analysis. Therefore, this variable was not included in the analysis, and we could not assess its relevance to the development of the severe COVID-19 disease course in our research.

Our results showed that systemic and lower respiratory symptoms were more commonly associated with severe COVID-19, while upper respiratory symptoms may be linked to milder illness. In the multivariable logistic regression analysis, febrile fever initially emerged as the only clinical variable associated with severe COVID-19. However, after internal validation of the model, this association was no longer statistically significant. These findings suggest that clinical symptoms at admission may have limited value as independent predictors of severe COVID-19. This observation is consistent with previous large-scale studies showing that commonly reported symptoms, such as fever, cough, or headache, do not independently predict COVID-19 severity (ICU admission or critical illness) after adjustment for confounding factors, highlighting the greater prognostic relevance of demographic characteristics and laboratory biomarkers [47–53]. Although dyspnoea has been identified as the symptom most consistently associated with severe outcomes in prior studies [47–53], it remains unclear whether symptoms such as shortness of breath should be considered independent predictors of severe COVID-19 or rather manifestations of ongoing clinical deterioration. Furthermore, the longer delay in hospital presentation among severe cases also suggested that early recognition and timely care could play a role in preventing disease progression. The finding that patients with severe COVID-19 were admitted to the hospital later after symptoms’ onset than those with non-severe illness (7 days vs. 4 days, respectively) raises an important question: whether more severe symptoms appear later in the course of the disease or whether the patients seek medical help later in the disease course [54–57]. There was a possibility that some patients with milder symptoms at first might not be admitted to the hospital immediately but would return later when their condition worsened. We did not evaluate the fact of revisits to the emergency department in our study; therefore, we could not answer this question. This possibility emphasizes how crucial it is to recognize early clinical features, relevant aspects of patient history, or laboratory-based warning signs, which might point to a higher risk of developing severe disease. Better tools for the early identification of high-risk patients could ensure they get the necessary care at the right time.

This study reinforces the clinical utility of widely available laboratory tests for early risk stratification in COVID-19. Our research results demonstrated that CRP, LDH, IL-6, AST, and ferritin, performed on admission, demonstrated good accuracy for predicting severe COVID-19 cases. Multivariable analysis confirmed lymphopenia, elevated levels of CRP and LDH, together with age and obesity, to be predictors for a severe COVID-19 course. An integrated approach that combines biomarkers of inflammation, activation of the coagulation cascade, organ injury, and immune suppression with simple clinical observations is consistent with recent initiatives to create straightforward, scalable assessment instruments for the early detection of at-risk patients, particularly in limited-resource or high-burden settings [7,49]. The associations of severe COVID-19 with CRP, IL-6, LDH, AST, and ALT reinforce the prevailing notion that systemic inflammation and hepatic involvement are crucial factors in the initial phases of illness progression. LDH, in particular, has been widely recognized as a robust marker of tissue damage and hypoxia in severe viral pneumonias. In COVID-19 patients, higher LDH levels were related to increased odds of developing severe disease [58], higher mortality, longer invasive mechanical ventilation [59], and ARDS development [60]. Similarly, increased CRP and IL-6 levels were found in severe COVID-19 cases, indicating an infectious-inflammatory response [61].

A lower lymphocyte count was one of the main haematological differences observed in patients with severe COVID-19 at the time of hospitalization, reflecting the commonly reported lymphopenia in advanced cases. Lymphopenia also remained an independent risk factor for severe COVID-19 disease course, increasing the odds 3.70 times (95% CI: 1.37–9.99). Analogous results were published in a meta-analysis of 13 case series, which found that lymphopenia increases the risk of severe COVID-19 disease 3-fold [62]. While this marker may not be sensitive enough for broad screening, it may serve as a valuable rule-in criterion that could help to confirm the likelihood of severe disease when present. Additional studies support this finding, showing that patients with lower lymphocyte counts were more likely to be admitted to the ICU and had longer hospital stays [63]. Ongoing lymphopenia has also been linked to a higher risk of developing ARDS [64], and when combined with T-cell suppression, it has been associated with more severe illness and worse clinical outcomes [20].

In addition to clinical history, imaging features like CT bilateral involvement, non-ground-glass opacities have emerged as strong predictors of disease severity and mortality [57]. When discussing chest CT findings, we found out that infiltration, peripheral opacity, and pleural effusion were significantly more common in severe COVID-19 cases. As chest CT was performed in only one-fourth of our cohort, we did not include CT findings in the final regression model and could not assess them as independent predictors.

A comparison of regional data from Central and Eastern Europe – an area still underrepresented in global COVID-19 research – adds important context and reinforces the key findings of our study. To contextualize our findings within our region, we reviewed Polish studies alongside a large Estonian population-based cohort. Though methodologically diverse, these regional datasets provide valuable insights into COVID-19 severity and its clinical predictors. A large retrospective cohort study from Estonia, using nationwide e-health records, analysed 66,295 individuals with confirmed COVID-19 and 254,958 population controls [65]. It found that male sex, older age, and comorbidity burden were strongly associated with disease severity, with renal disease, previous myocardial infarction, and obesity being the top contributors to critical illness. Predictors of mortality also included renal, liver, cerebrovascular disease, or cancer. Although this registry-based study did not assess clinical symptoms, its large-scale, anonymized dataset provides rare, population-level insights from the Baltic region. In Poland, a large single-centre cohort of 5,191 hospitalized patients found that diabetes was associated with higher rates of ICU admission and mortality [66]. Independent predictors of poor outcomes included chronic kidney disease, heart failure, elevated CRP, D-dimer, and hyperglycaemia, especially in patients with preexisting metabolic disorders. This aligns with the Estonian study’s emphasis on renal and cardiometabolic comorbidities but adds detail by including laboratory-based predictors. Another Polish study emphasized baseline comorbidities, such as COPD, hypertension, and diabetes, as predictors of severe progression, reinforcing the broader pattern observed across all datasets [67]. Together, these findings from Central and Eastern European cohorts complement our results by showing consistent risk profiles. Despite differences in sample size and scope, this underscores the added value of including clinical symptoms and laboratory biomarkers to enhance risk stratification in hospital settings.

Our previous study on COVID-19 in-hospital mortality, based on a larger depersonalized cohort from the same institution, identified several overlapping and distinct predictors of adverse outcomes [68]. Specifically, in-hospital mortality was significantly associated with age, obesity, congestive heart failure, COPD, previous stroke, and elevated concentrations of urea (> 7.01 mmol/L), LDH (> 452.5 U/L), CRP (> 92.68 mg/L), IL-6 (> 69.55, ng/L), troponin I (> 18.95 ng/L), and ALT to AST ratio (> 1.49). Notably, older age, obesity, CRP, and LDH were significantly associated with both severe disease progression and mortality, underlining their central role in COVID-19 pathophysiology.

Although our analysis did not include data on specific SARS-CoV-2 variants, placing the findings within the context of Lithuania’s COVID-19 wave timeline provides valuable insights into wave-specific variations in disease presentation. Lithuania’s initial wave (spring 2020) was driven by the wild‐type virus. During the study period, COVID-19 in Lithuania progressed through three distinct waves, dominated by the Alpha (until September 30, 2020), Beta (from October 1, 2020, to July 1, 2021), and Delta (until December 31, 2021) variants. Following our study period, Omicron and its sublineages (BA.1, BA.2, BA.4/BA.5) took hold in early 2022, marked by extremely high case numbers but consistently lower case severity and fewer ICU admissions compared to Delta. Our findings correspond with global observations and show that severe COVID‑19 rates were significantly higher in wave 2 (93.8%) and wave 3 (87.1%) compared with wave 1 (43.6%) in Lithuania. Throughout the pandemic, each successive wave often brought shifts in hospital admission criteria and treatment recommendations as clinicians adapted to the virulence of circulating viral variants and incorporated accumulated knowledge. Notably, later waves dominated by Beta and Delta variants tended to involve more selective hospitalization, often reserving inpatient care for only the most severe cases, which likely amplified the observed severity rates despite advances in treatment and prevention protocols [69–72]. Also, the Alpha and Delta waves were characterized by higher hospitalization and ICU admission rates, reflecting increased disease severity [71,73–75]. In contrast, the Omicron waves had reduced case severity, although the number of infections skyrocketed [76–78]. Therefore, while our results showed increased severity in later waves, this likely reflects a combination of biological factors – such as the greater virulence of the Delta variant – and health-care related factors, including stricter hospital admission driven by resource constraints and updated clinical guidelines.

Interestingly, although only a small proportion of the cohort was vaccinated, we observed a higher prevalence of vaccinated individuals in the severe disease group. In Lithuania, COVID-19 vaccination began on December 27, 2020, initially targeting healthcare workers. On March 26, 2021, eligibility expanded to include individuals with chronic illnesses and those aged 65 and older – groups inherently at greater risk of developing severe disease. Mass vaccination of the general population only commenced on May 31, 2021. As our study period largely overlapped with these early phases of the rollout, most vaccinated patients in our cohort likely belonged to high-risk groups predisposed to poorer outcomes. Similar patterns have been reported in other retrospective inpatient studies, where the protective effects of vaccination are confounded by the underlying vulnerability of early vaccine recipients, as well as by a lack of adjustment for timing, dose number, and immune response status [79]. Due to the small number of vaccinated individuals in our cohort, consistent with the limited vaccine availability during the early rollout phase, we could not perform detailed subgroup analyses or reliably assess the impact of vaccination on disease severity.

Altogether, our study underlines the multifactorial nature of severe COVID-19. The combination of older age, obesity, immune suppression, and organ dysfunction biomarkers defines a recognizable profile of patients at the highest risk for developing severe disease, which could support triage decisions and early treatment escalation in hospital settings. Furthermore, by contributing data from Lithuania, this study helps expand global understanding of COVID-19, particularly within the context of Central and Eastern Europe. These populations have been underrepresented in global research datasets, despite experiencing substantial burdens during the pandemic. Differences in healthcare infrastructure, vaccination strategies, and population characteristics may limit the applicability of risk prediction models developed in Western Europe, Asia, or North America to the Baltic region. Our findings thus offer important insights for regional decision-making and health system preparedness, while contributing to broader efforts toward international validation of risk prediction models.

### Strengths and limitations

A major strength of our work is the inclusion of comprehensive variables, including symptoms, laboratory analytes, and imaging, from a single centre with standardized protocols, which helps to minimize interinstitutional variability.

However, several limitations ought to be acknowledged. First, the study was conducted at a single tertiary care centre, which may limit the generalizability of the findings to other healthcare settings with differing patient populations, clinical practices, and resource availability. As a tertiary hospital with strict admission criteria, our centre may have treated a higher proportion of patients with more severe disease, particularly during the later pandemic waves.

The partially retrospective design may have introduced information bias, particularly due to undocumented comorbidities and subjective clinical symptoms. Smoking status was not systematically recorded for retrospectively included patients, which resulted in a substantial proportion of missing smoking data and limited our ability to reliably assess its association with COVID-19 severity. Although BMI data were available for a large proportion of the cohort, it was not complete for all participants. To ensure transparency and minimize misinterpretation, both obesity and malnutrition were assessed using all available documentation, including BMI values and diagnostic codes. Nonetheless, some degree of underreporting or misclassification may still be present.

Several laboratory variables had relatively high rates of missing data. Laboratory testing upon admission was performed according to clinical indications; therefore, more analytes were tested more frequently in patients whose condition was more severe at the time of admission. As a result, some comparisons involved smaller subgroups, and some predictors that showed significance in univariable logistic regression could not be included in the final multivariable model, potentially affecting the comprehensiveness of the analysis.

The study extended across several pandemic waves, during which clinical practice and hospital admission strategies evolved. In later waves, hospital admission was generally reserved for patients with more severe disease. This may have influenced the distribution of clinical characteristics and outcomes across pandemic waves. Finally, due to the absence of SARS-CoV-2 sequencing data, we could not analyse outcomes by specific viral variants. Instead, COVID-19 waves were classified based on their timing relative to known variant prevalence in Lithuania, which may limit the precision of wave-based comparisons.

### Conclusions

Our results contribute to the knowledge that various chronic comorbidities and increased levels of biomarkers of inflammation and tissue injury influence the risk of developing a severe COVID-19 disease course. We found that older age, obesity, lymphopenia, and higher CRP and LDH levels were associated with severe COVID-19 disease course. Combining relevant patient history with routinely available laboratory parameters provides a practical risk stratification approach to help clinicians identify high-risk patients early and intervene quickly and efficiently. This approach may not only improve individual outcomes but also ease the strain on healthcare systems, both during the COVID-19 pandemic and in future pandemics, particularly in regions where such research and data remain limited.

## Supporting information

S1 Table Predictors of severe COVID-19, univariable logistic regression analysis. ALT – alanine aminotransferase; AST – aspartate aminotransferase; CI – confidence interval; COPD – chronic obstructive pulmonary disease; CRP – C-reactive protein; IL-6 – interleukin 6; IQR – interquartile range; LDH – lactate dehydrogenase; NEWS – National Early Warning Score; NT-proBNP – N-terminal pro-B-type natriuretic peptide; OR – odds ratio; SpO₂ – oxygen saturation.
(PDF)

S2 Table Clinical symptoms (as reported by patients) of COVID-19 on admission by pandemic waves. (PDF)

S3 Table Laboratory characteristics of hospitalized COVID-19 patients on admission by pandemic waves. ALT – alanine aminotransferase; ALP – alkaline phosphatase; AST – aspartate aminotransferase; CRP – C-reactive protein; GGT – gamma-glutamyl transferase; IL-6 – interleukin 6; INR – international normalized ratio; IQR – interquartile range; LDH – lactate dehydrogenase; NT-proBNP – N-terminal pro-B-type natriuretic peptide; PT – prothrombin time; WBC – white blood cell.
(PDF)

S4 Table Instrumental investigation findings, oxygen therapy, complications, and results of hospitalization of COVID-19 patients by pandemic waves. ECMO – extracorporeal membrane oxygenation; HDU – high dependency unit; ICU – intensive care unit; IQR – interquartile range.
(PDF)

S5 Data Data file. (XLSX)
