# Supplementary material for: Predicting severe COVID-19 disease in adults: A single-centre cohort study during the first three pandemic waves in 2020–2021 in Vilnius, Lithuania
Source: PLoS One. 2026 May 29;21(5):e0350112. doi: 10.1371/journal.pone.0350112 (PMC13221065; doi:10.1371/journal.pone.0350112)
Supplement: S1 Table — ALT – alanine aminotransferase; AST – aspartate aminotransferase; CI – confidence interval; COPD – chronic obstructive pulmonary disease; CRP – C-reactive protein; IL-6 – interleukin 6; IQR – interquartile range; LDH – lactate dehydrogenase; NEWS – National Early Warning Score; NT-proBNP – N-terminal pro-B-type natriuretic peptide; OR – odds ratio; SpO₂ – oxygen saturation. (PDF) [file pone.0350112.s001.pdf]

Predictor	Univariable regression	
	OR (95% CI)	p-value
Age in years, median (IQR)	1.04 (1.03–1.06)	<0.001
Male sex	1.55 (1.08–2.22)	0.018
Obesity	3.49 (1.99–6.09)	<0.001
Pregnancy	0.58 (0.09–3.50)	0.548
Anaemia or any other haematological disease	1.50 (0.76–2.99)	0.246
Asthma	1.30 (0.47–3.56)	0.615
COPD	9.49 (1.23–73.15)	0.031
Other pulmonary diseases, not asthma or COPD	1.89 (0.50–7.23)	0.350
Non-haematological oncological disease	1.82 (0.78–4.21)	0.164
Arterial hypertension	2.11 (1.46–3.03)	<0.001
Other cardiovascular disease (not arterial hypertension)	1.83 (1.15–2.92)	0.011
Dementia	1.23 (0.36–3.49)	0.838
Diabetes mellitus	1.82 (0.99–3.36)	0.055
Immunodeficiency	2.88 (0.80–10.35)	0.105
Chronic liver disease, including cirrhosis	0.69 (0.27–1.77)	0.443
Neuromuscular disease	1.41 (0.42–4.76)	0.576
Chronic kidney disease	1.59 (0.74–3.43)	0.240
Rheumatological disease	1.71 (0.59–4.94)	0.320
Prior stroke	0.69 (0.27–1.77)	0.443
Subfebrile fever	1.58 (1.08–2.30)	0.018
Febrile fever	2.89 (1.94–4.32)	<0.001
Chills	2.13 (1.40–3.24)	<0.001
Tachypnoea	11.42 (5.16–25.30)	<0.001
Malaise	4.29 (2.73–6.74)	<0.001
Headache	0.86 (0.59–1.27)	0.462
Dizziness	3.96 (2.19–7.15)	<0.001
Confusion	2.80 (1.03–7.62)	0.044
Myalgia	0.89 (0.60–1.33)	0.570
Sore throat	0.65 (0.42–1.00)	0.051
Coryza	1.00 (0.60–1.65)	0.983
Cough	1.71 (1.16–2.52)	0.007
Shortness of breath	5.81 (3.78–8.92)	<0.001
Chest pain	2.09 (1.32–3.30)	0.002
Palpitation	1.55 (0.78–3.07)	0.210
General deterioration	2.92 (1.91–4.48)	<0.001
Nausea	1.44 (0.80–2.62)	0.227
Vomiting	1.41 (0.42–4.76)	0.576
Diarrhoea	2.40 (1.30–4.42)	0.005
Abdominal pain	1.08 (0.49–2.35)	0.851
Ageusia	1.48 (0.88–2.48)	0.140
Anosmia	1.04 (0.67–1.60)	0.866
Conjunctivitis	1.23 (0.36–4.26)	0.742
Rash/other dermatological manifestation	2.12 (0.22–20.47)	0.518
SpO_2	0.60 (0.53–0.67)	<0.001
NEWS	3.30 (2.51–4.29)	<0.001
Neutrophils	1.21 (1.10–3.33)	<0.001
Lymphocyte count	0.37 (0.26–0.51)	<0.001
Lymphopenia (lymphocytes $\leq 1.00 \times 10^9$/L)	3.47 (2.29–5.27)	<0.001
ALT	1.01 (1.01–1.02)	<0.001
AST	1.04 (1.03–1.05)	<0.001
Ferritin	1.00 (1.00–1.00)	<0.001
IL-6	1.01 (1.00–1.02)	0.001
LDH	1.01 (1.01–1.02)	<0.001
D-dimer	1.00 (1.00–1.00)	0.019
Fibrinogen	1.64 (1.36–1.97)	<0.001
CRP	1.03 (1.02–1.03)	<0.001
Lactate	1.60 (1.12–2.27)	0.009
Troponin I	1.00 (1.00–1.00)	0.902
NT-proBNP	1.00 (1.00–1.00)	0.849
Creatinine >	1.00 (1.00–1.01)	0.213
Urea	1.12 (1.03–1.21)	0.007
