# Supplementary material for: Predicting severe COVID-19 disease in adults: A single-centre cohort study during the first three pandemic waves in 2020–2021 in Vilnius, Lithuania
Source: PLoS One. 2026 May 29;21(5):e0350112. doi: 10.1371/journal.pone.0350112 (PMC13221065; doi:10.1371/journal.pone.0350112)
Supplement: S2 Table — (PDF) [file pone.0350112.s002.pdf]

Symptoms	Wave 1 (Alfa, until 30Sep2020, N=337)		Wave 2 (Beta, 1Oct2020 – 31Jul2021, N=96)		Wave 3 (Delta, 1Aug2021 – 31Dec2021, N=62)		p-value		
	Total	n (%)	Total	n (%)	Total	n (%)	Wave 1 vs wave 2	Wave 1 vs wave 3	Wave 2 vs wave 3
Sudden onset of symptoms	325	277 (85.2)	95	67 (70.5)	62	49 (79.0)	0.001	0.22	0.236
Subfebrile fever	337	218 (64.7)	96	68 (70.8)	62	40 (64.5)	0.262	0.979	0.404
Febrile fever	337	100 (29.7)	96	56 (58.3)	62	23 (37.1)	<0.001	0.245	0.009
Chills	332	77 (23.2)	96	32 (33.3)	62	34 (54.8)	0.045	<0.001	0.007
Tachypnoea	337	48 (14.2)	96	31 (32.3)	62	12 (19.4)	<0.001	0.301	0.074
Malaise	330	233 (70.6)	96	89 (92.7)	62	54 (87.1)	<0.001	0.007	0.24
Headache	330	100 (30.3)	96	24 (25.0)	62	28 (45.2)	0.314	0.022	0.008
Dizziness	330	30 (9.1)	96	30 (31.3)	62	25 (40.3)	<0.001	<0.001	0.242
Confusion	333	15 (4.5)	96	5 (5.2)	62	4 (6.5)	0.773	0.517	0.739
Myalgia	330	96 (29.1)	96	21 (21.9)	62	25 (40.3)	0.163	0.079	0.013
Sore throat	330	74 (22.4)	96	13 (13.5)	62	14 (22.6)	0.057	0.978	0.141
Coryza	330	37 (11.2)	96	21 (21.9)	62	15 (24.2)	0.007	0.006	0.734
Cough	331	210 (63.4)	96	75 (78.1)	62	56 (90.3)	0.007	<0.001	0.047
Shortness of breath	331	110 (33.2)	96	49 (51.0)	62	39 (62.9)	0.001	<0.001	0.143
Chest pain	330	69 (20.9)	96	22 (22.9)	62	23 (37.1)	0.673	0.006	0.054
Palpitations	330	16 (4.8)	96	10 (10.4)	62	15 (24.2)	0.045	<0.001	0.021
General deterioration	335	57 (17.0)	96	51 (53.7)	62	43 (69.4)	<0.001	<0.001	0.050
Nausea	330	22 (6.7)	96	17 (17.7)	62	15 (24.2)	<0.001	<0.001	0.322
Vomiting	337	5 (1.5)	96	3 (3.1)	62	4 (6.5)	0.292	0.036	0.434
Diarrhoea	333	32 (9.6)	96	14 (14.6)	62	16 (25.8)	0.165	<0.001	0.079
Abdominal pain	330	17 (5.2)	96	9 (9.4)	62	2 (3.2)	0.128	0.750	0.203
Ageusia	330	40 (12.1)	95	18 (18.9)	62	17 (27.4)	0.088	0.002	0.212
Anosmia	330	0 (0.0)	95	18 (21.2)	62	31 (50.0)	0.118	0.002	<0.001
Conjunctivitis	337	58 (17.6)	96	24 (25.3)	62	27 (43.5)	0.094	<0.001	0.017
Rash/other dermatological manifestation	337	6 (1.8)	96	2 (2.1)	62	3 (4.8)	1.000	0.15	0.381

χ^2 test or Fisher's exact test, as appropriate, was used for calculations.

Compared to COVID-19 pandemic wave 1, patients in wave 2 more frequently reported febrile fever (58.3% vs. 29.7%, $p < 0.001$), chills (33.3% vs. 23.2%, $p = 0.045$), tachypnoea (32.3% vs. 14.2%, $p < 0.001$), malaise (92.7% vs. 70.6%, $p < 0.001$), dizziness (31.3% vs. 9.1%, $p < 0.001$), coryza (21.9% vs. 11.2%, $p = 0.007$), cough (78.1% vs. 63.4%, $p = 0.007$), shortness of breath (51.0% vs. 33.2%, $p = 0.001$), palpitations (10.4% vs. 4.8%, $p = 0.045$), general deterioration (53.7% vs. 17.0%, $p < 0.001$), and nausea (17.7% vs. 6.7%, $p < 0.001$).

Compared to wave 1, patients in wave 3 more frequently reported chills (54.8% vs. 23.2%, $p < 0.001$), malaise (87.1% vs. 70.6%, $p = 0.007$), headache (45.2% vs. 30.3%, $p = 0.022$), dizziness (40.3% vs. 9.1%, $p < 0.001$), coryza (24.2% vs. 11.2%, $p = 0.006$), cough (90.3% vs. 63.4%, $p < 0.001$), shortness of breath (62.9% vs. 33.2%, $p < 0.001$), chest pain (37.1% vs. 20.9%, $p = 0.006$), palpitations (24.2% vs. 4.8%, $p < 0.001$), general deterioration (69.4% vs. 17.0%, $p < 0.001$), nausea (24.2% vs. 6.7%, $p < 0.001$), vomiting (6.5% vs. 1.5%, $p = 0.036$), diarrhoea (25.8% vs. 9.6%, $p < 0.001$), ageusia (27.4% vs. 12.1%, $p = 0.002$), anosmia (50.0% vs. 0.0%, $p = 0.002$), and conjunctivitis (43.5% vs. 17.6%, $p < 0.001$).

Compared to wave 2, patients in wave 3 more frequently reported chills (54.8% vs. 33.3%, $p = 0.007$), headache (45.2% vs. 25.0%, $p = 0.008$), myalgia (40.3% vs. 21.9%, $p = 0.013$), cough (90.3% vs. 78.1%, $p = 0.047$), palpitations (24.2% vs. 10.4%, $p = 0.021$), general deterioration (69.4% vs. 53.7%, $p = 0.050$), anosmia (50.0% vs. 21.2%, $p < 0.001$), and conjunctivitis (43.5% vs. 25.3%, $p = 0.017$), and less frequently – febrile fever (37.1% vs. 58.3%, $p = 0.009$).
