# Supplementary material for: Predicting severe COVID-19 disease in adults: A single-centre cohort study during the first three pandemic waves in 2020–2021 in Vilnius, Lithuania
Source: PLoS One. 2026 May 29;21(5):e0350112. doi: 10.1371/journal.pone.0350112 (PMC13221065; doi:10.1371/journal.pone.0350112)
Supplement: S3 Table — ALT – alanine aminotransferase; ALP – alkaline phosphatase; AST – aspartate aminotransferase; CRP – C-reactive protein; GGT – gamma-glutamyl transferase; IL-6 – interleukin 6; INR – international normalized ratio; IQR – interquartile range; LDH – lactate dehydrogenase; NT-proBNP – N-terminal pro-B-type natriuretic peptide; PT – prothrombin time; WBC – white blood cell. (PDF) [file pone.0350112.s003.pdf]

Analyte	Wave 1 (Alfa, until 30Sep2020, N=337)		Wave 2 (Beta, 1Oct2020 – 31Jul2021, N=96)		Wave 3 (Delta, 1Aug2021 – 31Dec2021, N=62)		p-value		
	Total	Median (IQR)	Total	Median (IQR)	Total	Median (IQR)	Wave 1 vs wave 2	Wave 1 vs wave 3	Wave 2 vs wave 3
WBC, $x10^9$/L	323	5.37 (4.19–6.90)	96	6.13 (4.89–7.59)	62	5.88 (4.28–7.55)	0.044	0.853	1.000
Neutrophils, $x10^9$/L	323	3.30 (2.42–4.80)	96	4.60 (3.47–6.09)	62	4.33 (3.12–5.73)	<0.001	0.003	1.000
Lymphocytes, $x10^9$/L	323	1.30 (0.94–1.73)	96	0.99 (0.78–1.28)	62	0.88 (0.69–1.31)	<0.001	<0.001	1.000
Platelets, $x10^9$/L	323	186.00 (149.00–232.00)	96	190.50 (149.50–243.00)	62	193.00 (147.00–247.25)	1.000	0.929	1.000
ALP, U/L	261	63.00 (52.00–76.11)	80	67.50 (53.00–98.25)	51	58.00 (43.00–102.00)	0.473	1.000	0.392
ALT, U/L	292	25.14 (16.00–42.09)	93	37.00 (24.00–66.00)	58	42.00 (22.00–84.00)	<0.001	<0.001	1.000
AST, U/L	280	29.00 (21.92–42.00)	93	38.00 (28.50–66.00)	57	60.00 (36.50–79.00)	<0.001	<0.001	0.027
Ferritin, μg/L	248	263.78 (126.30–505.96)	91	695.80 (329.00–1,374.00)	59	560.00 (260.00-1,543.00)	<0.001	<0.001	1.000
IL-6, ng/L	258	18.30 (8.30–35.85)	89	40.80 (21.85–68.95)	54	42.60 (25.50–65.13)	<0.001	<0.001	1.000
LDH, U/L	259	239.04 (190.35–295.00)	93	338.00 (272.15–427.00)	55	346.00 (251.00–468.00)	<0.001	<0.001	1.000
D-dimer, μg/L	244	302.50 (180.00–543.75)	91	500.00 (345.00–770.00)	60	430.00 (285.00–796.25)	<0.002	0.005	0.651
Fibrinogen, g/L	237	4.86 (3.89–5.75)	66	6.02 (4.99–6.75)	57	5.63 (4.88–6.45)	<0.001	<0.001	1.000
CRP, mg/L	322	14.63 (3.90–44.35)	95	84.00 (40.60–127.30)	62	93.55 (34.78–151.98)	<0.001	<0.001	1.000
Lactate, mmol/L	220	1.12 (0.88–1.69)	87	1.45 (1.14–1.87)	60	1.36 (1.06–2.18)	<0.001	0.006	1.000
Troponin I, ng/L	246	6.00 (2.75–12.00)	85	10.00 (6.15–17.94)	56	4.70 (2.85–8.00)	<0.001	0.485	<0.001
NT-proBNP*, ng/L*	211	113.20 (60.50–375.70)	81	182.00 (95.60–364.00)	47	209.00 (123.00–544.00)	0.006	0.038	1.000
GGT, IU/L	258	25.00 (15.00–56.00)	81	63.00 (35.50–128.00)	52	61.00 (24.25–113.00)	<0.001	<0.001	1.000
PT, %	246	88.00 (75.50–100.00)	83	91.00 (83.00–110.00)	58	98.50 (83.75–112.00)	0.002	0.080	0.612
INR	251	1.05 (1.00–1.12)	84	1.04 (0.96–1.08)	58	1.00 (0.96–1.07)	0.001	0.351	0.110
Creatinine, μmol/L	309	78.09 (64.20–95.21)	96	74.50 (64.00–92.75)	62	77.25 (63.40–96.90)	1.000	1.000	1.000
Urea, mmol/L	228	4.70 (3.52–6.49)	86	4.44 (3.50–6.39)	62	4.85 (3.88–6.23)	1.000	1.000	1.000

Kruskal–Wallis test was used for the analysis.
