# Supplementary material for: Predicting severe COVID-19 disease in adults: A single-centre cohort study during the first three pandemic waves in 2020–2021 in Vilnius, Lithuania
Source: PLoS One. 2026 May 29;21(5):e0350112. doi: 10.1371/journal.pone.0350112 (PMC13221065; doi:10.1371/journal.pone.0350112)
Supplement: S4 Table — ECMO – extracorporeal membrane oxygenation; HDU – high dependency unit; ICU – intensive care unit; IQR – interquartile range. (PDF) [file pone.0350112.s004.pdf]

Characteristic	Wave 1 (Alfa, until 30Sep2020, N=337)	Wave 2 (Beta, 1Oct2020 – 31Jul2021, N=96)	Wave 3 (Delta, 1Aug2021 – 31Dec2021, N=62)	p-value		
				Wave 1 vs wave 2	Wave 1 vs wave 3	Wave 2 vs wave 3
**Chest X-ray**	320 (95.0)	94 (97.9)	59 (95.2)	0.269	1.000	0.381
Infiltration	208 (65.2)	91 (96.8)	55 (93.2)	<0.001	<0.001	0.430
Pleural effusion	25 (7.8)	2 (2.1)	4 (6.8)	0.050	1.000	0.206
Infiltration opacity > 50%	18 (9.9)	14 (40.0)	13 (46.4)	<0.001	<0.001	0.608
Stasis	46 (14.4)	9 (9.6)	3 (5.1)	0.228	0.051	0.372
**Chest computed tomography**	102 (30.3)	15 (15.6)	9 (14.5)	-	-	-
Peripheral opacity	47 (46.1)	12 (80.0)	6 (66.7)	0.014	0.306	0.635
Ground-glass opacity	61 (59.8)	12 (80.0)	9 (100.0)	0.132	0.025	0.266
Consolidation	20 (19.6)	7 (46.7)	4 (44.4)	0.043	0.100	1.00
Infiltration	39 (38.2)	14 (93.3)	9 (100.0)	<0.001	<0.001	1.000
Pleural effusion	10 (9.8)	4 (26.7)	1 (11.1)	0.081	1.000	0.615
**Pneumonia**	240 (74.3)	95 (99.0)	60 (96.8)	<0.001	<0.001	0.562
Unilateral	48 (14.9)	8 (8.3)	4 (6.5)	<0.001	<0.001	1.000
Bilateral	192 (59.4)	87 (90.6)	56 (90.3)			
**Oxygen therapy**	147 (43.6)	90 (93.8)	54 (87.1)	<0.001	<0.001	0.151
Nasal cannula	59 (17.5)	12 (12.5)	16 (25.8)	0.242	0.124	0.032
Face mask	93 (27.6)	81 (84.4)	44 (71.0)	<0.001	<0.001	0.043
High-flow therapy	2 (0.6)	3 (3.1)	9 (14.5)	0.075	<0.001	0.012
Non-invasive ventilation	3 (0.9)	0 (0.0)	2 (3.2)	1.000	0.174	0.152
Mechanical ventilation	8 (2.4)	2 (2.1)	4 (6.5)	1.000	0.099	0.212
ECMO	2 (0.6)	0 (0.0)	1 (1.6)	1.000	0.398	0.392
**Complications**						
Any complication	64 (19.0)	33 (34.4)	18 (29.0)	0.001	0.072	0.483
Acute respiratory distress syndrome	30 (8.9)	5 (5.2)	11 (17.7)	0.241	0.035	0.011
Bronchiolitis	1 (0.3)	0 (0.0)	0 (0.0)	1.000	1.000	-
Secondary bacterial pneumonia (confirmed by positive bronchoalveolar lavage culture)	6 (1.8)	2 (2.1)	2 (3.2)	1.000	0.360	0.646
Other secondary bacterial infection (confirmed by positive microbiological cultures)	32 (9.5)	20 (20.8)	8 (12.9)	0.003	0.412	0.202
Sepsis	12 (3.6)	2 (2.1)	4 (6.5)	0.744	0.289	0.212
Acute renal failure	11 (3.3)	4 (4.2)	3 (4.8)	0.751	0.464	1.000
Heart failure	4 (1.2)	0 (0.0)	1 (1.6)	0.580	0.572	0.392
Multiple organ dysfunction	11 (3.3)	0 (0.0)	3 (4.8)	0.133	0.464	0.059
Dermatological complications	2 (0.6)	1 (1.0)	0 (0.0)	0.529	1.000	1.000
Critical illness myopathy	1 (0.3)	0 (0.0)	0 (0.0)	1.000	1.000	-
Pulmonary embolism	2 (0.6)	4 (4.2)	2 (3.2)	0.024	0.116	1.000
Other complication	15 (4.5)	6 (6.3)	6 (6.3)	0.430	0.115	0.541
Transferred to ICU or HDU	26 (7.7)	15 (15.6)	13 (21.0)	0.020	0.001	0.390
Transferred to ICU	26 (7.7)	10 (10.4)	13 (21.0)	0.398	0.001	0.066
Transferred to HDU	0 (0.0)	9 (9.4)	2 (3.2)	<0.001	0.024	0.203
**Length of stay**						
Length of stay in hospital in days, median (IQR)	11 (7–14)	10 (8–15)	9 (8–14)	0.232	0.179	0.558
Length of stay in ICU in days, median (IQR)	7.5 (4.5–14)	7 (3–11.5)	7 (5–16)	0.548	0.990	0.588
Length of stay in HDU in days, median (IQR)	7.5 (4.5–14)	10 (8–14)	7 (5–16)	0.434	0.792	0.665
Length of stay in ICU and HDU in days, median (IQR)	15 (9–28)	14 (8–25)	14 (10–32)	0.762	0.93	0.708
**Hospital discharge status**						
Discharged / Transferred to other hospital	302 (89.6)	92 (95.8)	58 (93.5)	0.137	0.531	0.078
Died	16 (4.7)	1 (1.0)	4 (6.5)			

Mann-Whitney U test, χ^2 test, or Fisher's exact test, as appropriate, were used for calculations.
